# Supplementary material for: Employee education, labor protection intensity and auditor risk perception
Source: PLoS One. 2024 Jun 21;19(6):e0298938. doi: 10.1371/journal.pone.0298938 (PMC11192339; doi:10.1371/journal.pone.0298938)
Supplement: S1 Appendix — (DOCX) [file pone.0298938.s001.docx]

**S1 Appendix. The process of enacting and amending the Labor Protection Law in China.**

The enactment and amendment process of the Labor Protection Law in China followed the legislative procedures of the National People’s Congress and its Standing Committee. The development and revisions of these regulations reflect the Chinese government’s concern for safeguarding workers’ rights, promoting social stability, and supporting economic development. The process of enacting and amending the Labor Protection Law in China occurred mainly in the 1990s. Here is the timeline and key events:

1. July 5 1994: The Standing Committee of the National People’s Congress approved the "Labor Law of the People’s Republic of China" and announced that it would officially take effect on January 1, 1995.

https://www.gov.cn/banshi/2005-05/25/content_905.htm

2.June 29, 2007：The “Labor Law” underwent several revisions over the years to adapt to changes in society and the economy. One significant amendment was the promulgation of the “Labor Contract Law of the People’s Republic of China” in 2007. The 28th session of the Standing Committee of the Tenth National People’s Congress deliberated and adopted, and on January 1, 2008, the Labor Contract Law of the People’s Republic of China was implemented, commonly known as the new “Labor Protection Law”. The new “Labor Protection Law” further improves the labor contract system.

The legislative guiding ideology of the Labor Law is: (1) Fully embody the principles of the Constitution, highlighting the protection of workers’ rights and interests; (2)It is conducive to promoting the development of productive forces; (3) To stipulate uniform basic standards and norms; (4)Adhere to the national conditions of our country, as far as possible with the international practice.

https://www.gov.cn/gongbao/content/2007/content_711013.htm?ivk_sa=1024320u

3.December 28, 2012：Decision of the Standing Committee of the National People’s Congress on Amending the Labor Contract Law of the People’s Republic of China, adopted at the 30th Session of the Standing Committee of the Eleventh National People’s Congress of the People’s Republic of China on December 28, 2012, is hereby promulgated and shall come into force as of July 1, 2013.The newly revised Labor Contract Law on July 1, 2013 focuses on solving the problem of labor dispatch.

https://www.gov.cn/flfg/2012-12/28/content_2305571.htm
