# Supplementary figures and images for: Employee education, labor protection intensity and auditor risk perception
Source: PLoS One. 2024 Jun 21;19(6):e0298938. doi: 10.1371/journal.pone.0298938 (PMC11192339; doi:10.1371/journal.pone.0298938)

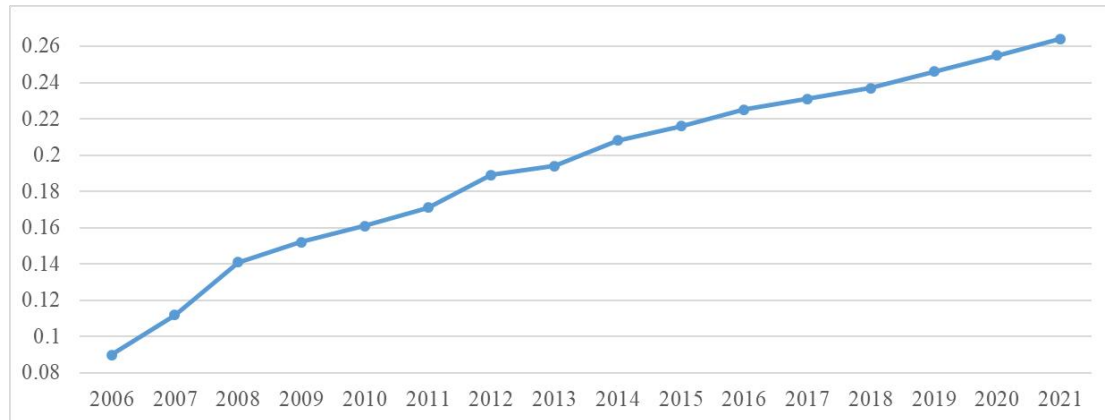

**Fig 1. Employee Education Level**

Supplement: S1 Fig — (PDF) [file pone.0298938.s002.pdf]
