# Supplementary material for: Employee education, labor protection intensity and auditor risk perception
Source: PLoS One. 2024 Jun 21;19(6):e0298938. doi: 10.1371/journal.pone.0298938 (PMC11192339; doi:10.1371/journal.pone.0298938)
Supplement: Attachment — Submitted filename: Review_PONE-D-23-32966.pdf [file pone.0298938.s003.pdf]

Review for the Manuscript Number PONE-D-23-32966

Title: Employee Education, Labor Protection Intensity and Auditor Risk Perception

The paper examines the impact of employee education degree on financial audit fees moderated by the passage of the Labor Protection Law. The authors found that such impact is negative, suggesting a reduction of the audit fees in case of low marketization level and weak Confucian culture intensity; however, this effect is significantly reduced or totally offset by the Labor Protection Law.

Overall, I think the paper is very interesting and adds to the literature; the research question is sufficiently novel due to the scant evidence on employee education degree and financial auditing.
That said, the paper would benefit from some deep revisions in terms of readability. Another crucial dimension is to outline the main policy implications arising from the study: what can investors, managers, and institutions learn?
I think the paper is worth it, therefore, I urge the authors to address the points raised in this report.

Comments:

1. Authors claim that hand-collected data of employees' education level. Why did this happen? Is important to figure out that this manuscript is submitted to an international journal and different readers of different countries have different knowledge. E.g., in the EU, information about employees' education degree is mandatory; every year firms must release such information, which is compiled in an official database. It would be interesting to know if the authors did not have access to such dataset, or if simply it does not exist in China.
2. Once, and using the same arguments as before, It would be interesting to learn more about the passage of the Labor Protection Law in China. Might be as an appendix (tip: search in papers about the effects of EU directives enactment).
3. Lines 56-61. This sentence must be reformulated because ideas are too messy: “At present, existing studies (…)
4. Lines 174-175. The authors cite Dechow and Dichev (2002) to support the following idea: “Previous studies have shown that in terms of mandatory information disclosure, higher employee education (…)”; the work of Dechow and Dichev (2002) does not perfectly fit in the spirit of such statement. I suggest finding a different study to support it.
5. I suggest simplifying hypothesis 1. E:g. “Employees with high educational background can effectively reduce the audit fees borne by the company.”
6. In the “Descriptive statistics” section, avoid the expression “certain”. It is not accurate and does not match the scientific method spirit. Perhaps it could be replaced by “suggests”.
7. In Table 4, variables LNAF and SIZE should be presented in monetary units.
8. Also find another expression to “Hypothesis (1) 2 has been verified”; e.g., “evidence supports Hypothesis (1) 2”.
9. At this point, I’m wondering if auditor rotation is mandatory in China. I suggest some clarification about that issue.
10. And about the employee director system in China, is employee-board representation mandatory? Do you anticipate any impact on controlling for that in your current results?

11. The section “Sensitivity test of estimation method” could be included as a footnote because it does not really add anything new.
12. The “Mediation effect test” section is confusing because the number of Tables is wrong (see Minor Errors) and repeating the same equations is confusing. I suggest elaborating more on the rationale behind this analysis.
13. A gap in this study is the absence of coefficients’ estimates interpretation. For example, the interaction coefficients in Table 6 should be interpreted. If one sums EDU+ LAW+ EDU×LAW, one will find a positive value, meaning the passage of the Law surpasses the EDU effect.

Minor Issues:

- Revise the number of Tables. E.g: line 353 refers to Table 4, but the paragraph reports to Table 6.
- Acronyms such as CSMAR, ST and *ST should be described.
- Lines 80/81: In this sentence: “Past research mainly focuse on the audit background of the executive (…)” the right form is “focuses”.
- References 36 and 37 are repeated.
- Line 190: Spells “whistleblowing”.
- Line 235: In this sentence: “Based on this, this paper puts forward the following hypotheses”, is not hypotheses but hypothesis.
