# Supplementary material for: Employee education, labor protection intensity and auditor risk perception
Source: PLoS One. 2024 Jun 21;19(6):e0298938. doi: 10.1371/journal.pone.0298938 (PMC11192339; doi:10.1371/journal.pone.0298938)
Supplement: Attachment — Submitted filename: Response to Reviewers.docx [file pone.0298938.s004.docx]

**Manuscript Title:** Employee Education, Labor Protection Intensity and Auditor Risk Perception

**Manuscript ID:** PONE-D-23-32966

**Response to** **Editor and Reviewer**

Dear Editor and Reviewer,

We are most grateful to you for giving us another opportunity to revise and resubmit our paper. We would like to express our deep gratitude towards you for the clear, constructive, and insightful comments that have helped us further improve the manuscript. We took full account of all points that you raised and have revised the paper accordingly. We believe that the quality of the manuscript, as a whole, has been greatly improved by these revisions, which we hope will resolve and address your concerns.

Below we explain how we respond to your comments, detailing our revisions and clarifications. To ensure that the comments and our responses are clearly identified, the former are shown in ***italicised blue* *text***, and our response to each comment is given underneath the queries in black.

With all best wishes,

Authors

**Our general responses:**

We thank you so much for your comments on our work. Your suggestions were vital to further enhance the development of our paper and improve its overall contribution. We have, as explained below, carefully and thoughtfully addressed your concerns in this revised manuscript.

**Journal requirements Comment 1**

*When submitting your revision, we need you to address these additional requirements. Please ensure that your manuscript meets PLOS ONE’s style requirements, including those for file naming. The PLOS ONE style templates can be found at*

*https://journals.plos.org/plosone/s/file?id=wjVg/PLOSOne_formatting_sample_main_body.pdf and*

*https://journals.plos.org/plosone/s/file?id=ba62/PLOSOne_formatting_sample_title_authors_affiliations.pdf.*

**Responses:**

We thank you very much for your constructive comments and suggestions. We are very sorry for our negligence of style requirements, we have made correction format according to the Reviewer’ s comments.

**Journal requirements Comment 2**

*In your Data Availability statement, you have not specified where the minimal data set underlying the results described in your manuscript can be found. PLOS defines a study’s minimal data set as the underlying data used to reach the conclusions drawn in the manuscript and any additional data required to replicate the reported study findings in their entirety. All PLOS journals require that the minimal data set be made fully available. For more information about our data policy, please see http://journals.plos.org/plosone/s/data-availability.*

*Upon re-submitting your revised manuscript, please upload your study’s minimal underlying data set as either Supporting Information files or to a stable, public repository and include the relevant URLs, DOIs, or accession numbers within your revised cover letter. For a list of acceptable repositories, please see http://journals.plos.org/plosone/s/data-availability#loc-recommended-repositories. Any potentially identifying patient information must be fully anonymized.*

*Important: If there are ethical or legal restrictions to sharing your data publicly, please explain these restrictions in detail. Please see our guidelines for more information on what we consider unacceptable restrictions to publicly sharing data: http://journals.plos.org/plosone/s/data-availability#loc-unacceptable-data-access-restrictions. Note that it is not acceptable for the authors to be the sole named individuals responsible for ensuring data access.*

*We will update your Data Availability statement to reflect the information you provide in your cover letter.*

**Responses:**

We have re-written this part according to the Reviewer’ s suggestion.

Data Availability: The data used in this study are third-party data owned by CSMAR (China Stock Market & Accounting Research Database), therefore, the authors have no right to share the data. Interested persons can contact CSMAR for the data (see https://data.csmar.com/ for more details, contact via email: service@csmar.com, tel: 400-639-8883). The authors confirm that they did not have any special access or privileges to the data that other researchers would not have.

**Additional Editor Comments**

*Dear Authors, After careful consideration and review, we request major amendments to this manuscript before accepting it for publication. Please answer the reviewers comments to improve the manuscript, as it has area to improve in all section, introduction, hypothesis development, methodology and conclusion. Here are few tips and some recommended paper that will help you to revise the manuscript.*

*Please address the issues raised by the reviewers regarding the theoretical part of hypothesis development. Moreover, there is an important strand of literature with respect to the Audit Quality in following studies. These studies will help you to improve the methodology as well.*

*ESG, Dividend Payout Policy and Audit Quality: An empirical Evidence from the Western Europe*

*Borsa Istanbul Review*

*https://doi.org/10.1016/j.bir.2022.10.012*

*The role of Audit Quality in ESG-Corporate Financial Performance nexus: empirical evidence from western European companies*

*Borsa Istanbul Review*

*https://doi.org/10.1016/j.bir.2022.08.011*

*Moderating role of audit quality in ESG performance and capital financing dynamics: insights in China. Environ Dev Sustain (2023). https://doi.org/10.1007/s10668-023-03636-9*

*Make sure to proofread the manuscript before it is resubmitted to the journal. Please go through the journal’s guidelines thoroughly and revise the paper accordingly. Thank you for submitting your paper to the PlosOne.*

**Responses:**

We thank you very much for your constructive comments and suggestions. We have added the above important literature to our research. For details, please refer to the revised manuscript. We have added the following literature based on your valuable comments and those of the editorial department:

“CEO’s duality negatively affects a firm’s financial performance.”

Ali S, Naseem M A, Jiang J, Rehman R U, Malik F, Ahmad M I. “How” and “When” CEO duality matter? Case of a developing economy[J]. SAGE Open, 2022, 12(3): 21582440221116113.

“Economic complexity in China had been found to have a statistically significant impact on the country’s ecological footprint.”

Ali S, Jiang J, Hassan S T, Shah A A. Revolution of nuclear energy efficiency, economic complexity, air transportation and industrial improvement on environmental footprint cost: a novel dynamic simulation approach[J]. Nuclear Engineering and Technology, 2022, 54(10): 3682-3694.

“There is a significant positive correlation between ESG and dividends.”

Zahid R M A, Taran A, Khan M K, Chersan I C. ESG, dividend payout policy and the moderating role of audit quality: empirical evidence from Western Europe[J]. Borsa Istanbul Review, 2023, 23(2): 350-367.

“Firms with better ESG performance found to have less debt financing and easier access to equity capital from stock markets. However, the results did not show a significant impact of audit quality on this relationship.”

Zahid R M A, Khan M K, Anwar W, Maqsood U S. The role of audit quality in the ESG-corporate financial performance nexus: Empirical evidence from Western European companies[J]. Borsa Istanbul Review, 2022, 22: S200-S212.

“ESG has a significantly negative effect on a firm’s financial performance as measured by the return on assets (ROA), supporting the trade-off hypothesis in which investing in ESG activities increases the cost of business.”

Zahid R M A, Saleem A, Maqsood U S, Sági Judit. Moderating role of audit quality in ESG performance and capital financing dynamics: insights in China[J]. Environment, Development and Sustainability, 2023: 1-30.

**Reviewer #1 Comment 1**

*I was pleased to read the article Employee Education, Labor Protection Intensity and Auditor Risk Perception. This study is needed. However, there are a number of issues that need to be addressed. An abstract should describe the purpose or need of the study.*

**Responses:**

We thank you very much for your constructive comments and suggestions. We have revised and supplemented the abstract. For the convenience of your review, we have listed here:

“Prior literature finds senior executives can influence auditor decision making. However, few studies have discussed the impact of employee’s personal characteristics. Our research aims to fill the above research gaps by examining the impact of employee level education on audit costs. Taking A-share listed companies in Shanghai and Shenzhen from 2006 to 2021 as the research object, this paper examines the impact of employee education on audit fees. It is found that highly educated employees can effectively reduce the audit fees borne by the company, but the implementation of the Labor Protection Law weakens this inhibitory effect. In the case of low marketization level and weak Confucian culture intensity, employee education level has a more significant inhibitory effect on audit fees of listed companies. This study provides a basis for empirical research on the impact of employee attributes on auditor decision making, provides a new research perspective on the impact of labor protection law at the corporate micro level, and enriches the theoretical research on corporate governance rooted in traditional Chinese culture. We contribute to the practice that implications for evaluating the effectiveness of adopting labor protection.”

**Reviewer #1 Comment 2**

*In my opinion, the introduction section was very lengthy. This section explains the study’s context and objective. The research gap should also be narrowed after analyzing previous studies. The research method is not adequately explained in the first section.*

**Responses:**

Thank you very much for your constructive comment and insight. We have revised the introduction according to your valuable comments. For the convenience of your review, we have listed here:

“Economic complexity in China has been shown to significantly impact the country’s ecological footprint (Ali et al., 2022). The nationvs strategy of rejuvenation through science, education, and the cultivation of qualified personnel has notably elevated the education level of the Chinese population. The resulting demographic quality dividend is gradually compensating for the diminishing demographic quantity dividend, offering enduring support for sustained economic growth (Han, 1999). Demographic quality is closely linked to the stable economic growth and the overarching rejuvenation of the nation. As such, it has become a shared concern among enterprises, government entities, and the academic community. Consequently, the level of education has emerged as a crucial indicator for measuring the human capital within enterprises (Schultz, 1961). Rajan and Zingales (1998) formalized the human capital theory of corporate governance, asserting that the governance challenge is no longer confined to the upper echelons of the corporate power structure but must extend to all employee levels. Presently, studies predominantly concentrate on the impact of management education characteristics, including enterprise investment behavior (Jiang et al., 2009), diversified decision-making (Dyreng et al., 2010), market participation (Xiao and Zhang, 2012), determination of subjective social status (Andersson, 2018), and entrepreneurship quality (Eesley and Lee, 2021). While some research, based on questionnaire surveys, investigates the influence of all employees’ education level characteristics on wage levels (Zhan et al., 2012) and total profits (Cheng et al., 2016), limited attention has been directed towards exploring whether the characteristics of all employees impact corporate governance and auditors’ decisions. Audit fees, serving as a direct reflection of the auditor’s risk estimation for the auditee, convey crucial signals to accounting information users. The study of audit fees is paramount for assessing audit market competitiveness and probing issues related to contracting and independence in the audit process (Hay et al., 2006). Thus, a critical question arises: Do auditors believe that the education level of all employees diminishes audit risk by enhancing the quality of financial statements, leading to reduced audit fees? Additionally, how does the heightened intensity of labor protection mandated by the Labor Protection Law either foster or hinder the influence of highly educated employees on audit fees?

Based on this, our research aims to fill the above research gaps by examining the impact of employee level education on audit costs. This paper uses manually collected data on employee education level of Shanghai and Shenzhen A-share listed companies from 2006 to 2021 to measure employee education level, and tests whether the implementation of the Labor Protection Law can strengthen or inhibit the effect of highly educated employees on enterprise audit fees. Our findings reveal that companies with higher employee education levels tend to have lower audit fees compared to those with lower education levels. Moreover, we observe that labor protection significantly diminishes the inhibitory effect of highly educated employees on audit fees. Through a series of robustness tests, these findings persist. Further analysis indicates that the inhibitory impact of employee education on audit expenses is more pronounced in companies operating in less marketized environments and with weaker Confucian cultural influences.

The contribution of this paper is as follows: Firstly, this paper expands the research scope of the impact of employee attributes on auditor decision making. Past research mainly focuses on the audit background of the executive (Cai et al., 2015), the military experience of executives (Quan et al., 2018), the accent of executives (Yuan et al., 2018), background characteristics of executive management team (Chen and Zhou, 2016), behavioral integrity of CEO (Dikolli et al, 2020) for auditors decision-making. This paper’s significance lies in its use of manually collected data on employees’ education levels, providing an accurate depiction of the educational qualifications of all employees within an enterprise. It affirms that the personal characteristics of employees beyond senior executives can indeed exert an influence on auditor decision-making.

Secondly, from the perspective of employees, this paper directly studies the effect of the implementation of the Labor Protection Law on the individual perspective of employees. Existing studies mainly explore the impact of the implementation of the Labor Protection Law on investment efficiency (Lu et al., 2015) and business elasticity (Liao et al., 2014) from the enterprise level, and economic growth from the region level (Xu et al, 2022). This paper introduces a novel research perspective from the employees’ standpoint, shedding light on the impact of the implementation of the Labor Protection Law at the micro-level of enterprises.

Finally, our study is confined to a single country (China), we acknowledge our findings may not generalize to other regions due to institutional differences (Wong 2016). Therefore, it is possible that the introduction of labor protection do not has the same effect on the auditing process in other countries. Unfortunately, we are unable to test our hypotheses in other countries because the required employee’ education level data are unavailable. Even so, our study has implications for regions where labor protection are being considered for adoption or have been introduced. Meanwhile, when assessing the effectiveness of implementing labor protection measures, regulators should place increased emphasis on the perception of audit risk.”

**Reviewer #1 Comment 3**

*The results of this study are not supported by significant and recent literature, although they are relevant and have been adequately discussed. Additionally, the most recent research articles should be cited. Please refer to https://doi.org/10.1016/j.net.2022.05.022; as <https://doi.org/10.1177/21582440221116113>*

**Responses:**

We thank you very much for your constructive comments and suggestions. We have added the above important literature to our research. For details, please refer to the revised manuscript. We have added the following literature based on your valuable comments and those of the editorial department:

“CEO’s duality negatively affects a firm’s financial performance.”

Ali S, Naseem M A, Jiang J, Rehman R U, Malik F, Ahmad M I. “How” and “When” CEO duality matter? Case of a developing economy[J]. SAGE Open, 2022, 12(3): 21582440221116113.

“Economic complexity in China had been found to have a statistically significant impact on the country’s ecological footprint.”

Ali S, Jiang J, Hassan S T, Shah A A. Revolution of nuclear energy efficiency, economic complexity, air transportation and industrial improvement on environmental footprint cost: a novel dynamic simulation approach[J]. Nuclear Engineering and Technology, 2022, 54(10): 3682-3694.

“There is a significant positive correlation between ESG and dividends.”

Zahid R M A, Taran A, Khan M K, Chersan I-C. ESG, dividend payout policy and the moderating role of audit quality: empirical evidence from Western Europe[J]. Borsa Istanbul Review, 2023, 23(2): 350-367.

“Firms with better ESG performance found to have less debt financing and easier access to equity capital from stock markets. However, the results did not show a significant impact of audit quality on this relationship.”

Zahid R M A, Khan M K, Anwar W, Maqsood U S. The role of audit quality in the ESG-corporate financial performance nexus: Empirical evidence from Western European companies[J]. Borsa Istanbul Review, 2022, 22: S200-S212.

“ESG has a significantly negative effect on a firm’s financial performance as measured by the return on assets (ROA), supporting the trade-off hypothesis in which investing in ESG activities increases the cost of business.”

Zahid R M A, Saleem A, Maqsood U S, Sági Judit. Moderating role of audit quality in ESG performance and capital financing dynamics: insights in China[J]. Environment, Development and Sustainability, 2023: 1-30.

**Reviewer #2 Comment 1**

*Authors claim that hand-collected data of employees’ education level. Why did this happen? Is important to figure out that this manuscript is submitted to an international journal and different readers of different countries have different knowledge. E.g., in the EU, information about employees’ education degree is mandatory; every year firms must release such information, which is compiled in an official database. It would be interesting to know if the authors did not have access to such dataset, or if simply it does not exist in China.*

**Responses:**

Thank you for your valuable comments. In China, the education level of employees is disclosed in the annual financial statement. When we started this study, there was no database on the education level of all employees, so we collected these data manually from the financial statements of listed Chinese companies. However, at present, the CSMAR database has updated the education level database of all employees of listed companies in China, which is completely consistent with the data collected manually by us after verification.

https://data.csmar.com

**Reviewer #2 Comment 2**

*Once, and using the same arguments as before, It would be interesting to learn more about the passage of the Labor Protection Law in China. Might be as an appendix (tip: search in papers about the effects of EU directives enactment).*

**Responses:**

Thank you very much for your constructive comment and insight. The enactment and amendment process of the Labor Protection Law in China followed the legislative procedures of the National People’s Congress and its Standing Committee. The development and revisions of these regulations reflect the Chinese government’s concern for safeguarding workers’ rights, promoting social stability, and supporting economic development. We can visit the official website of the National People’s Congress, which may provide official texts of laws and regulations as well as related legal processes and documents. The web address are http://www.npc.gov.cn/ and https://www.gov.cn/

The process of enacting and amending the Labor Protection Law in China occurred mainly in the 1990s. We have included this part in the appendix of the article. Here is the timeline and key events:

1. July 5 1994: The Standing Committee of the National People’s Congress approved the "Labor Law of the People’s Republic of China" and announced that it would officially take effect on January 1, 1995.

https://www.gov.cn/banshi/2005-05/25/content_905.htm

2.June 29, 2007：The “Labor Law” underwent several revisions over the years to adapt to changes in society and the economy. One significant amendment was the promulgation of the “Labor Contract Law of the People’s Republic of China” in 2007. The 28th session of the Standing Committee of the Tenth National People’s Congress deliberated and adopted, and on January 1, 2008, the Labor Contract Law of the People’s Republic of China was implemented, commonly known as the new “Labor Protection Law”. The new “Labor Protection Law” further improves the labor contract system.

The legislative guiding ideology of the Labor Law is: (1) Fully embody the principles of the Constitution, highlighting the protection of workers’ rights and interests; (2)It is conducive to promoting the development of productive forces; (3) To stipulate uniform basic standards and norms; (4)Adhere to the national conditions of our country, as far as possible with the international practice.

https://www.gov.cn/gongbao/content/2007/content_711013.htm?ivk_sa=1024320u

3.December 28, 2012：Decision of the Standing Committee of the National People’s Congress on Amending the Labor Contract Law of the People’s Republic of China, adopted at the 30th Session of the Standing Committee of the Eleventh National People’s Congress of the People’s Republic of China on December 28, 2012, is hereby promulgated and shall come into force as of July 1, 2013.The newly revised Labor Contract Law on July 1, 2013 focuses on solving the problem of labor dispatch.

https://www.gov.cn/flfg/2012-12/28/content_2305571.htm

**Reviewer #2 Comment 3**

*Lines 56-61. This sentence must be reformulated because ideas are too messy: “At present, existing studies (…)*

**Responses:**

Thank you very much for your valuable advice. We are very sorry that our wrong written expression caused your misunderstanding. Combined with the revision comments of other reviewers, the original sentence has been adjusted to lines 59-65 of the revised draft after modification. For your convenience, the following is the revised original sentence:

Presently, studies predominantly concentrate on the impact of management education characteristics, including enterprise investment behavior (Jiang et al., 2009), diversified decision-making (Dyreng et al., 2010), market participation (Xiao and Zhang, 2012), determination of subjective social status (Andersson, 2018), and entrepreneurship quality (Eesley and Lee, 2021). While some research, based on questionnaire surveys, investigates the influence of all employees’ education level characteristics on wage levels (Zhan et al., 2012) and total profits (Cheng et al., 2016), limited attention has been directed towards exploring whether the characteristics of all employees impact corporate governance and auditors’ decisions.

**Reviewer #2 Comment 4**

*Lines 174-175. The authors cite Dechow and Dichev (2002) to support the following idea: “Previous studies have shown that in terms of mandatory information disclosure, higher employee education (…)”; the work of Dechow and Dichev (2002) does not perfectly fit in the spirit of such statement. I suggest finding a different study to support it.*

**Responses:**

Thank you very much for your valuable comments. We are very sorry that our wrong understanding of the literature has caused your confusion. We incorporated revisions in response to your feedback, and you can review the detailed changes in the revised manuscript. For your convenience, we have also listed here:We cite another paper to support this part of our logic.

“Previous studies have shown that firms with a high-quality workforce exhibit higher accruals quality, fewer internal control violations (Call et al., 2017).”

Call A C, Campbell J L, Dhaliwal D S, et al. Employee quality and financial reporting outcomes[J]. Journal of Accounting and Economics, 2017, 64(1): 123-149.

**Reviewer #2 Comment 5**

*I suggest simplifying hypothesis 1. E:g. “Employees with high educational background can effectively reduce the audit fees borne by the company.”*

**Responses:**

Thank you for your invaluable feedback. The outstanding writing skills demonstrated in your insightful suggestions have significantly enhanced the readability of this article. We incorporated revisions in response to your feedback, and you can review the detailed changes in the revised manuscript. For your convenience, we have also listed here:

H1: Employees with high educational background can effectively reduce the audit fees borne by the company.

**Reviewer #2 Comment 6**

*In the “Descriptive statistics” section, avoid the expression “certain”. It is not accurate and does not match the scientific method spirit. Perhaps it could be replaced by “suggests”.*

**Responses:**

Thank you for your invaluable feedback. The outstanding writing skills demonstrated in your insightful suggestions have significantly enhanced the readability of this article. We incorporated revisions in response to your feedback, and you can review the detailed changes in the revised manuscript.

**Reviewer #2 Comment 7**

*In Table 4, variables LNAF and SIZE should be presented in monetary units.*

**Responses:**

Thank you very much for your constructive comment and insight. We annotate the currency unit RMB of LNAF and SIZE in Table 3. Besides the statistical significance of the data, the economic significance of the data is further marked in the paper. For your convenience, we have also listed here:

**Table 3 Variable Definitions**

| Variable | Variable Definition |
| --- | --- |
| *LNAF* | Natural logarithm of audit fees in RMB. |
| *SIZE* | Natural logarithm of the company’s total assets in RMB. |

“Table 4 reports the descriptive statistics results of each variable. It can be found that the mean, minimum and maximum value of audit fee (LNAF) are 13.6855, 12.0695 and 16.4759. This indicates that the actual values of the audit fees are approximately 808,151.68, 174,166.91, and 13,399,485.33 RMB, respectively, which are close to the study of Zhang et al. (2018).

In terms of control variables, the mean and median value of the size of listed company (SIZE) are 22.0631 and 21.9184. This suggests that the actual values for the company size are approximately 1,093,556,776.7 and 826,429,158.5 RMB, indicating that A-share listed companies exhibit a large size.”

**Reviewer #2 Comment 8**

*Also find another expression to “Hypothesis (1) 2 has been verified”; e.g., “evidence supports Hypothesis (1) 2”.*

**Responses:**

Thank you for your invaluable feedback. The outstanding writing skills demonstrated in your insightful suggestions have significantly enhanced the readability of this article. We incorporated revisions in response to your feedback, and you can review the detailed changes in the revised manuscript.

**Reviewer #2 Comment 9**

*At this point, I’m wondering if auditor rotation is mandatory in China. I suggest some clarification about that issue.*

**Responses:**

In China, different types of enterprises have different requirements for auditor rotation. We have sorted out the past auditor rotation system and the current auditor rotation system respectively:

**Auditor rotation policy in the past**

**For Chinese listed companies:** In October 2003, the China Securities Regulatory Commission and the Ministry of Finance jointly issued the Provisions on the Regular Rotation of Signature Certified Public Accountants in Securities and Futures Business (hereinafter referred to as the "Regular Rotation Provisions"), which mandated the regular rotation of signature certified public accountants in securities market audit business, and stipulated that under normal circumstances, signature certified public accountants should continuously provide audit for a certain relevant institution The total service shall not exceed five years.

Due to the long time and the document is now abolished, I could not find the source document page on the official website, the following is a reprint of the regulation on the unofficial website.

http://www.elinklaw.com/zsglmobile/lawView.aspx?id=50009

**Current policy on auditor rotation**

Because the accounting firm rotation policy needs to be perfected. Various state-owned enterprises have different practices and different implementation scales, and it is urgent to form a unified and standardized rotation system to achieve a reasonable balance between the rotation period and audit costs and audit risks. On February 20, 2023, the Ministry of Finance of China, the State-owned Assets Supervision and Administration Commission of the State Council and the Securities Regulatory Commission of the People’s Republic of China promulgated the "Administrative Measures for the Selection and Employment of Accounting Firms by State-owned Enterprises and Listed Companies", which stipulates:

In principle, a state-owned enterprise shall not continuously employ the same accounting firm for more than eight years. If a state-owned enterprise intends to continue to employ the same accounting firm for more than 8 years due to business needs, it shall comprehensively consider the audit quality, shareholders, evaluation, and opinions of regulatory authorities of the accounting firm in the early stage, and may appropriately extend the term of employment after fulfilling the corporate governance procedures and internal decision-making procedures, but the continuous term of employment shall not exceed 10 years.

Where a partner of an audit project or a signed certified public accountant has actually undertaken the audit business of the same state-owned enterprise or listed company for a cumulative period of five years, he shall not participate in the audit business of the state-owned enterprise or listed company for five consecutive years thereafter.

Where a partner of an audit project or a signed certified public accountant undertakes an initial public offering of shares or a public offering of shares to an unspecified party and lists audit services, the period for continuously performing audit services after listing shall not exceed two years.

https://www.gov.cn/zhengce/zhengceku/2023-05/05/content_5754176.htm

**Reviewer #2 Comment 10**

*And about the employee director system in China, is employee-board representation mandatory? Do you anticipate any impact on controlling for that in your current results?*

**Responses:**

Thank you very much for your constructive comment and insight. We have added the control variables related to employees and directors, and carried out the regression again, and the regression results are basically unchanged from the original regression. For your convenience, we have also listed the relevant content of employee director representation here:

**Table 1	Variable Definitions**

| Variable | Variable Definition |
| --- | --- |
| *StaffDirector* | The value is 1 if the company includes staff representatives within its board of directors, and 0 otherwise. |

**Table 2 Descriptive Statistics**

| Variable | N | Mean | S.D. | Min | P25 | Median | P75 | Max |
| --- | --- | --- | --- | --- | --- | --- | --- | --- |
| *StaffDirector* | 31748 | 0.0089 | 0.0938 | 0.0000 | 0.0000 | 0.0000 | 0.0000 | 1.0000 |

The mean value of staff representative directors (*StaffDirectoris*) is 0.0089, indicating that 0.89% of companies have staff representative directors.

As for the requirements of the employee director system, according to Article 44, paragraph 2 of the Company Law, limited liability companies established by two or more state-owned enterprises or other state-owned investment entities shall have employee representatives in the board of directors. For other limited liability companies, there may be employee representatives in the board of directors, but it is not mandatory. In addition, the China Securities Regulatory Commission has also put forward some requirements for independent directors of listed companies in the “Rules for Independent Directors of Listed Companies”, but it does not explicitly require listed companies to establish employee directors.

On January 5, 2022, the China Securities Regulatory Commission announced the Rules for Independent Directors of Listed Companies.

http://www.csrc.gov.cn/csrc/c101954/c1719987/content.shtml

Members of the board of directors may include representatives of the employees of the company, and the articles of association of the company shall specify whether the board of directors of the company may be represented by representatives of the employees, and the number of representatives of the employees as directors. The employees’ representatives on the board of directors shall be directly elected to the board of directors by the employees of the company through a democratic election of the employees’ congress, the general assembly of the employees or any other form.

On January 5, 2022, the China Securities Regulatory Commission announced the Guidelines on the Articles of Association of Listed Companies (Revised in 2022).

http://www.csrc.gov.cn/csrc/c101954/c1719264/content.shtml

**Reviewer #2 Comment 11**

*The section “Sensitivity test of estimation method” could be included as a footnote because it does not really add anything new.*

**Responses:**

Thank you very much for your suggestion, we have made adjustments according to your suggestions.

**Reviewer #2 Comment 12**

*The “Mediation effect test” section is confusing because the number of Tables is wrong (see Minor Errors) and repeating the same equations is confusing. I suggest elaborating more on the rationale behind this analysis.*

**Responses:**

We are very sorry that the mistake in our writing has caused your confusion. We have fixed our existing Minor Errors. Thank you very much for your valuable advice.

By testing the mediating effect, we hope that the quality of financial reports can play an intermediary role between the educational level of employees and the audit cost of listed companies, so as to strengthen the logical relationship between the educational level of all employees and the audit cost.

**Reviewer #2 Comment 13**

*A gap in this study is the absence of coefficients’ estimates interpretation. For example, the interaction coefficients in Table 6 should be interpreted. If one sums EDU+ LAW+ EDU×LAW, one will find a positive value, meaning the passage of the Law surpasses the EDU effect.*

**Responses:**

Thank you for your invaluable feedback. The outstanding writing skills demonstrated in your insightful suggestions have significantly enhanced the readability of this article. We incorporated revisions in response to your feedback, and you can review the detailed changes in the revised manuscript. For your convenience, we have also listed here:

Table 5 reports the regression results of employee education level and audit fees. If employee education level (*EDU*) is a negative value, meaning employees with high educational background can effectively reduce the audit fees borne by the company.

Table 6 reports the regression results of employee education level, labor protection intensity and audit fees. If the summation of *EDU*+*LAW*+*EDU*×*LAW* yields a positive value, indicating that the enactment of the Law surpasses the effect of EDU.

**Reviewer #2 Comment 14**

*Minor Issues:*

*- Revise the number of Tables. E.g: line 353 refers to Table 4, but the paragraph reports to Table 6.*

*- Acronyms such as CSMAR, ST and *ST should be described.*

*- Lines 80/81: In this sentence: “Past research mainly focuse on the audit background of the executive (…)” the right form is “focuses”.*

*- References 36 and 37 are repeated.*

*- Line 190: Spells “whistleblowing”.*

*- Line 235: In this sentence: “Based on this, this paper puts forward the following hypotheses”, is not hypotheses but hypothesis.*

**Responses:**

We are very sorry that the mistake in our writing has caused your confusion. We have fixed our existing Minor Errors. Thank you for your invaluable feedback. The outstanding writing skills demonstrated in your insightful suggestions have significantly enhanced the readability of this article. We incorporated revisions in response to your feedback, and you can review the detailed changes in the revised manuscript.
