# Supplementary material for: Employee education, labor protection intensity and auditor risk perception
Source: PLoS One. 2024 Jun 21;19(6):e0298938. doi: 10.1371/journal.pone.0298938 (PMC11192339; doi:10.1371/journal.pone.0298938)
Supplement: Attachment — Submitted filename: Response to Reviewers.docx [file pone.0298938.s005.docx]

**Manuscript Title:** Employee Education, Labor Protection Intensity and Auditor Risk Perception

**Manuscript ID:** PONE-D-23-32966R1

**Response to** **Editor and Reviewer**

Dear Editor and Reviewer,

We are most grateful to you for giving us another opportunity to revise and resubmit our paper. We would like to express our deep gratitude towards you for the clear, constructive, and insightful comments that have helped us further improve the manuscript. We took full account of all points that you raised and have revised the paper accordingly. We believe that the quality of the manuscript has been greatly improved by these revisions, which we hope will resolve and address your concerns.

Below we explain how we respond to your comments, detailing our revisions and clarifications. To ensure that the comments and our responses are clearly identified, the former are shown in ***italicised blue* *text***, and our response to each comment is given underneath the queries in black.

With all best wishes,

Authors

*1. If the authors have adequately addressed your comments raised in a previous round of review and you feel that this manuscript is now acceptable for publication, you may indicate that here to bypass the “Comments to the Author” section, enter your conflict of interest statement in the “Confidential to Editor” section, and submit your "Accept" recommendation.*

**Reviewer #2:** All comments have been addressed

*2. Is the manuscript technically sound, and do the data support the conclusions?
The manuscript must describe a technically sound piece of scientific research with data that supports the conclusions. Experiments must have been conducted rigorously, with appropriate controls, replication, and sample sizes. The conclusions must be drawn appropriately based on the data presented.*

**Reviewer #2:** Yes

*3. Has the statistical analysis been performed appropriately and rigorously?*

**Reviewer #2:** Yes

*4. Have the authors made all data underlying the findings in their manuscript fully available?
The [PLOS Data policy](http://www.plosone.org/static/policies.action#sharing) requires authors to make all data underlying the findings described in their manuscript fully available without restriction, with rare exception (please refer to the Data Availability Statement in the manuscript PDF file). The data should be provided as part of the manuscript or its supporting information, or deposited to a public repository. For example, in addition to summary statistics, the data points behind means, medians and variance measures should be available. If there are restrictions on publicly sharing data—e.g. participant privacy or use of data from a third party—those must be specified.*

**Reviewer #2:** Yes

*5. Is the manuscript presented in an intelligible fashion and written in standard English?
PLOS ONE does not copyedit accepted manuscripts, so the language in submitted articles must be clear, correct, and unambiguous. Any typographical or grammatical errors should be corrected at revision, so please note any specific errors here.*

**Reviewer #2:** Yes

*6. Review Comments to the Author*

***Reviewer #2:*** *Minor Issues:
- Include a footnote describing succinctly the auditor rotation process in China
- Revise the number of Tables. E.g: Revise lines 257/258
- Line 216: In this sentence: “Based on this, this paper puts forward the following hypotheses”, replace hypotheses per hypothesis.*

**Responses:**

We thank you very much for your constructive comments and suggestions. We are sorry that we have neglected some details, and we have made modifications according to your suggestions.

*7. PLOS authors have the option to publish the peer review history of their article ([what does this mean?](https://journals.plos.org/plosone/s/editorial-and-peer-review-process#loc-peer-review-history)). If published, this will include your full peer review and any attached files.
If you choose “no”, your identity will remain anonymous but your review may still be made public.
Do you want your identity to be public for this peer review? For information about this choice, including consent withdrawal, please see our [Privacy Policy](https://www.plos.org/privacy-policy).*

**Reviewer #2:** No
